# Single molecule TPM analysis of the catalytic pentad mutants of Cre and Flp site-specific recombinases: contributions of the pentad residues to the pre-chemical steps of recombination

**DOI:** 10.1093/nar/gkv114

**Published:** 2015-03-12

**Authors:** Hsiu-Fang Fan, Yong-Song Cheng, Chien-Hui Ma, Makkuni Jayaram

**Affiliations:** 1 Department of Life Sciences and Institute of Genome Sciences, National Yang Ming University, Taipei 112, Taiwan; 2 Department of Molecular Biosciences, University of Texas at Austin, Austin, TX 78712, USA

## Abstract

Cre and Flp site-specific recombinase variants harboring point mutations at their conserved catalytic pentad positions were characterized using single molecule tethered particle motion (TPM) analysis. The findings reveal contributions of these amino acids to the pre-chemical steps of recombination. They suggest functional differences between positionally conserved residues in how they influence recombinase-target site association and formation of ‘non-productive’, ‘pre-synaptic’ and ‘synaptic’ complexes. The most striking difference between the two systems is noted for the single conserved lysine. The pentad residues in Cre enhance commitment to recombination by kinetically favoring the formation of pre-synaptic complexes. These residues in Flp serve a similar function by promoting Flp binding to target sites, reducing non-productive binding and/or enhancing the rate of assembly of synaptic complexes. Kinetic comparisons between Cre and Flp, and between their derivatives lacking the tyrosine nucleophile, are consistent with a stronger commitment to recombination in the Flp system. The effect of target site orientation (head-to-head or head-to-tail) on the TPM behavior of synapsed DNA molecules supports the selection of anti-parallel target site alignment prior to the chemical steps. The integrity of the synapse, whose establishment/stability is fostered by strand cleavage in the case of Flp but not Cre, appears to be compromised by the pentad mutations.

## INTRODUCTION

Tyrosine site-specific recombinases comprise a large family of phosphoryl transfer enzymes that perform DNA exchange between well-defined target sites in a two-step reaction via a Holliday junction intermediate (1,2). The family name derives from the invariant active site tyrosine nucleophile, which executes strand cleavage by a transesterification mechanism analogous to that of type IB topoisomerases. Tyrosine recombinases are abundant among prokaryotes but were thought to be nearly absent among eukaryotes, except within the small budding yeast lineage Saccharomycetaceae. However, the DIRS and PAT families of retrotransposons and the presumed DNA transposons classified as Cryptons, identified in a large number of eukaryotes, harbor tyrosine recombinases (3,4). The sub-family of yeast tyrosine recombinases is coded for by plasmids related to the 2 micron circle of *Saccharomyces cerevisiae* (5,6).

The biological consequences of site-specific recombination are quite varied and encompass the choice between lysogenic and lytic pathways of phage development, resolution of chromosome dimers to promote equal segregation of phage, plasmid and bacterial genomes, and copy number maintenance in yeast plasmids (7–11). A subset of the well-characterized tyrosine recombinases, phage λ Int, phage P1 Cre, XerC/XerD from *Escherichia coli* and Flp from *S*. *cerevisiae*, have provided the templates for understanding the biochemical, structural and dynamic aspects of the strand cleavage and exchange steps of recombination (2,8,9,12,13). The ability to express and regulate Cre and Flp, and to a degree λ Int, in a wide variety of organisms has transformed them into biotechnological tools for engineering genomes into which the respective target sites have been incorporated (14–18).

**Figure 1.**
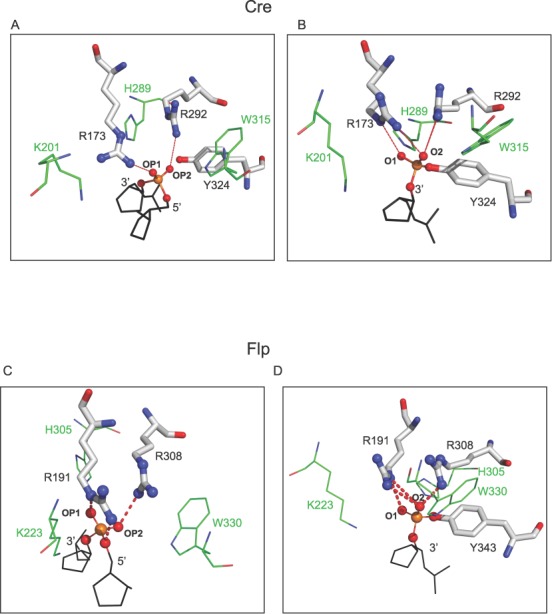
The arrangement of the catalytic pentad within the active sites of Cre and Flpe. **(A**, **B)** The distributions of the pentad residues within the Cre active sites containing the uncleaved DNA backbone (PDB: *4CRX*) (A) and the cleaved tyrosyl intermediate (PDB: *1CRX*) (B) are shown (22). **(C**, **D)** The Flp active site configurations with the uncleaved (PDB: *1M6X*) (C) and cleaved (PDB:*1M6X*) (D) DNA strand are displayed (32). The conserved arginine duo and their contacts with the non-bridging oxygen atoms of the scissile phosphodiester bond are highlighted. The conserved lysine, histidine and tryptophan residues are shown less prominently to avoid lack of clarity due to crowding. OP1 and OP2 refer to the pro-*S* and pro-*R* oxygen atoms of the scissile phosphodiester position. They are named O1 and O2, respectively, in the phosphotyrosyl intermediate. Note that the phosphate configuration is inverted as a result of cleavage. In the vanadate transition state structure of Cre (19), the oxygen atom contacted by Arg-292 is labeled as O1 (as opposed to O2 in this figure).

The active sites of tyrosine recombinases harbor, in addition to the tyrosine nucleophile, a conserved pentad of catalytic residues comprised of two arginines, a lysine, a histidine and either a histidine or a tryptophan (2). In addition, the negatively charged side chain of a conserved aspartic/glutamic acid indirectly helps catalysis by contributing to the structural integrity of the active site (19). In Cre, the pentad corresponds to Arg-173, Lys-201, His-289, Arg-292 and Trp-315 (Figure 1A and B). The corresponding residues in Flp are Arg-191, Lys-223, His-305, Arg-308 and Trp-330 (Figure 1C and D). Flp differs from Cre and other well-characterized tyrosine recombinases in assembling a shared active site by the donation of the cleavage nucleophile (Tyr-343) from one monomer to the pro-active site assembled by a neighboring monomer (20,21). As a result, strand cleavage by Flp occurs in *‘trans*’. By contrast, Cre, whose active site is housed entirely within a monomer, executes strand cleavage in *cis*. Consistent with the common chemistry of recombination shared by them, the active sites of Cre and Flp display very similar interactions of the catalytic residues with the scissile phosphate group (2,21–23) (Figure 1). They perform recombination *in vitro* under simple reaction conditions and are not selective with respect to the orientation of the target sites. They act on head-to-head (inverted) sites to bring about DNA inversion and on head-to-tail (direct) sites to cause DNA excision.

Single molecule tethered particle motion (TPM) analysis is well suited for characterizing the individual steps of the recombination pathway from start to finish (24–26). The Brownian motion (BM) amplitude of a polystyrene bead attached to a tethered DNA molecule (as measured by TPM) is exquisitely sensitive to the length of the tether, and reports faithfully on the pre-chemical and chemical steps of recombination that change the effective length of a DNA substrate. Recent TPM analyses of the Cre and Flp recombination reactions have revealed unexpected differences, against a backdrop of general similarities, between the two systems (24,25). While the recombinase-target site associations are rapid and efficient for both recombinases, the level of commitment to the productive reaction path is stronger for Flp than Cre. Strand cleavage by the tyrosine nucleophile appears to promote synapse formation by the Flp target sites (*FRT*s), but not by the Cre target sites (*loxP*s). Holliday junctions are transient during the Flp reaction, while they are encountered at a reasonable frequency during the Cre reaction. This observation is consistent with a slow step following Holliday junction isomerization detected by an analysis combining single molecule tethered fluorophore motion with fluorescence energy transfer (TFM-FRET) (27). The differences between Cre and Flp could, in principle, arise from the *cis* versus *trans* assembly of their active sites, subtle variations in the functional roles of their catalytic residues, non-equivalence in the conformational attributes of the two reactions or from a combination of these factors.

The present study, characterizing catalytic pentad variants of Cre and Flp, reveals the contributions of these residues toward the kinetic and thermodynamic attributes of the Cre and Flp reaction pathways.

## MATERIALS AND METHODS

### Proteins

The Cre mutants were expressed and purified according to published protocols (28). The wild-type Flp protein used in the assays was the more thermostable variant Flpe, containing four amino acid substitutions: Pro2Ser, Leu33Ser, Tyr108Asn and Ser294Pro (29). Flpe and its derivatives were purified for TPM assays as described previously (25). In describing specific experimental observations, we have referred to the relevant proteins as Flpe or Flpe with the appropriate mutation indicated in parenthesis. However, in more general contexts, we have replaced the designation Flpe by Flp.

### DNA substrates

The DNA substrates for the TPM analyses of Cre and Flpe mutants were the same as those described in a published work (24,25). The *loxP* sites were present within linear 1267 bp DNA molecules with a spacing of 619 bp between their crossover points. The locations of the *loxP* sites and the inter-*loxP* distance were the same for the substrates containing the sites in head-to-tail and head-to-head orientations. Analogous *FRT*-containing DNA substrates were 1168 bp long with a spacing of 687 bp between the two *FRT* sites.

### Single molecule TPM measurements and data analysis

The experimental conditions and the details of TPM measurements for the Cre-*loxP* and Flpe-*FRT* systems have been published previously (24,25). DNA molecules were conjugated to biotin at one 5′ end and to digoxigenin at the other. The digoxigenin-end of each molecule was attached to an anti-digoxigenin-coated cover slip and the biotin-end to a streptavidin-coated 200 nm polystyrene bead. The analysis, performed at room temperature (22°C), was initiated by adding Cre or Flpe or a mutant protein in the reaction buffer [50 mM Tris-HCl, pH = 7.5; 33 mM NaCl; 10 mM MgCl_2_; 5 mM dithiothreitol; 1 mg/ml bovine serum albumin]. The concentration of Cre or a Cre mutant in the reaction was 50 nM; that of Flp or a Flp mutant was 200 nM. Reactions were quenched at 30 min from the start by flowing in ∼100 μl of the reaction buffer containing 0.05% sodium dodecyl sulfate (SDS).

The concentrations of Cre or Flpe (or their pentad mutants) were well above the *K*_d_ values estimated for the wild-type proteins (30,31). The percentage of substrate molecules that associated with the recombinase is mentioned for each protein under ‘Results’ and listed in Supplementary Table S1. The fraction of a given type of pre-chemical complex (Table 1) was normalized to the recombinase-bound fraction of DNA molecules.

**Table 1.**
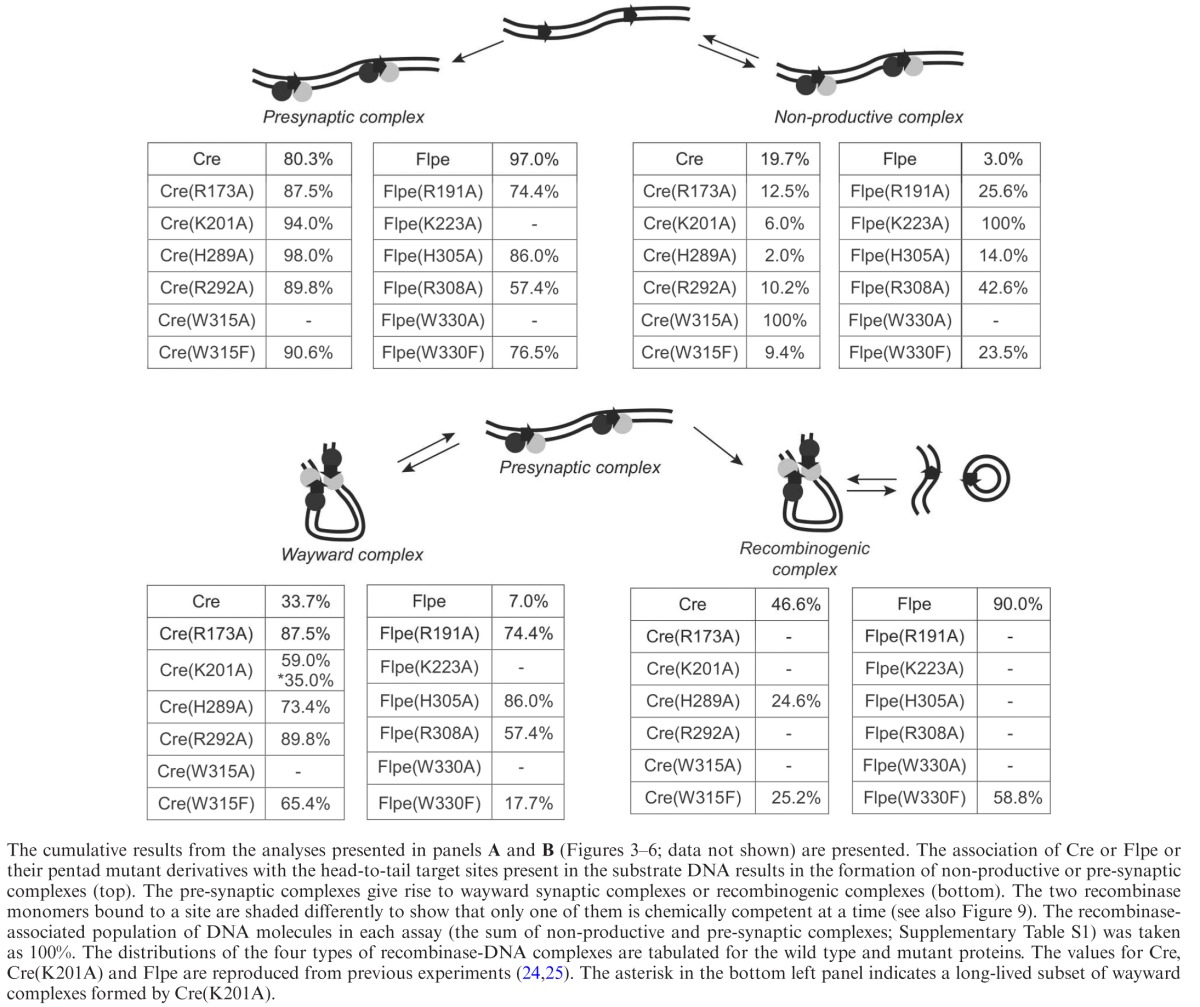
Relative fractions of distinct recombinase-target site complexes formed by Cre, Flpe and their variants mutated in the catalytic pentad residues

For deriving kinetic constants, the dwell time histograms were fitted to a single exponential algorithm, *y* = *A*_1_ x e^(-k^_1_^t)^, or to a double exponential algorithm, *y* = *A*_1_ x e^(-k^_1_^t)^ + *A*_2_ x e^(-k^_2_^t)^, where *A*_1_, *A*_2_, *k*_1_ and *k*_2_ are the fitting parameters determined by Origin 8.0. The fitting algorithm for obtaining each kinetic parameter has been explained in previous publications (24,25). The *R*^2^ values, indicating the goodness of fit, for the entire set of estimated rate constants were 0.93≤ *R*^2^ ≤ 1.00.

The rate constant for recombination by Flpe or a Flpe variant was derived from a DNA substrate containing head-to-head *FRT* sites. The dwell times of synapsed molecules prior to their decay to higher amplitude were fitted to a double exponential model to obtain the rate constant for the dissociation of wayward complexes and that for the DNA inversion reaction. For Cre or a Cre variant, the double exponential fit was applied to analogous dwell times of molecules containing *loxP* sites in head-to-head or head-to-tail orientation. In the latter case, the smaller rate constant represents the resolution of the Holliday junction intermediate in the parental mode (or recombination in the reverse direction).

## RESULTS

### The rationale of the TPM analysis

**Figure 2.**
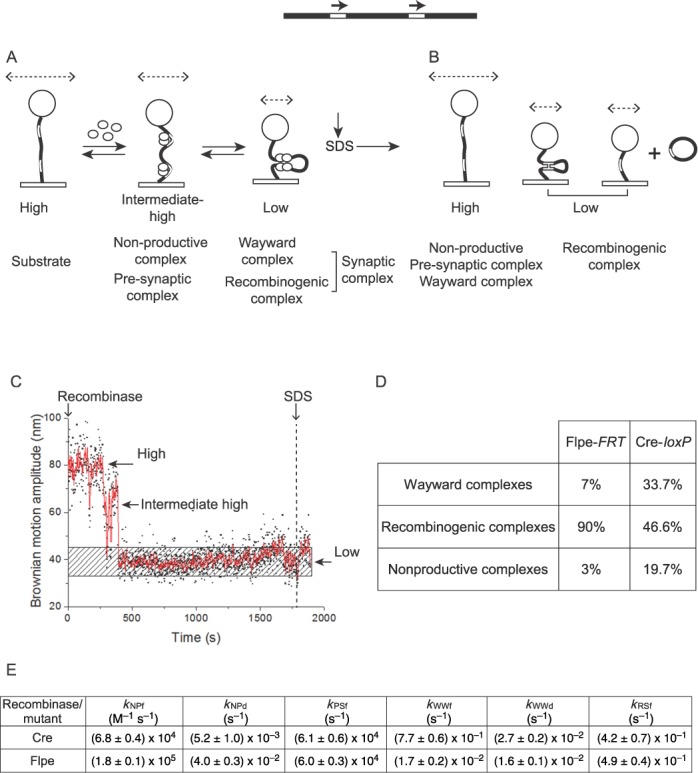
TPM analysis of site-specific recombination. **(A)** In the schematic diagram of the substrate DNA molecule, the recombination sites are shown as two open rectangular boxes, with the arrows above them indicating their head-to-tail orientation. The DNA is tethered to a coverslip at one end and to a polystyrene bead (drawn as a sphere) at the other. The events following the addition of the recombinase (shown as globules) and the corresponding changes in the BM amplitude of the bead (indicated by the lengths of the dashed lines with arrowheads at either end) are schematically outlined. The ‘high’ BM amplitude of the substrate switches to ‘intermediate high’ or ‘low’ depending on the state of recombinase-DNA association. The relevant complexes are termed non-productive, pre-synaptic and synaptic, and are described in the text in more detail. The synaptic complexes can be subdivided into two types, wayward or recombinogenic. **(B)** After treatment with SDS, only those molecules that have undergone strand exchange to form the Holliday junction intermediate or the excision products (signifying the recombinogenic complex) will remain in the low BM amplitude state. Molecules representing the non-productive, pre-synaptic or wayward complex will return to the high amplitude state. **(C)** In the time trace of a molecule, the three BM amplitude states are indicated, with the stippled bar denoting synapsis. This molecule formed stable synapsis early in the time course, and completed one or both strand exchange steps (as revealed by SDS addition at 30 min). **(D)** The relative abundance of non-productive, wayward and recombinogenic complexes estimated for the Cre-*loxP* and Flp-*FRT* systems from prior TPM analyses are tabulated (24,25). **(E)** The estimated rate constants for the pre-chemical steps of recombination are listed for wild-type Flpe and Cre (24,25). The corresponding data for their pentad mutants are given in Tables 2 and 3, respectively. NP = Non-productive complex. PS = Pre-synaptic complex. WW = Wayward synaptic complex. RS = Recombinogenic synaptic complex. The suffixes ‘f’ and ‘d’ denote the formation and dissociation of a complex, respectively.

The rationale of the TPM analysis (24–26) is outlined in Figure 2 with respect to two recombination sites in head-to-tail orientation, representing an excision reaction. There are three relevant BM amplitudes that signify the reaction stages of interest to us (Figure 2A–C). The tethered DNA substrate molecules prior to recombinase binding are in the high BM amplitude state, followed by recombinase-bound molecules with bent recombination sites (intermediate-high BM amplitude). The DNA molecules in which the sites are paired to form a synapse have low BM amplitude, which matches the BM amplitude of the Holliday junction intermediate or of the linear product of excision. When the protein is dissociated from DNA by SDS treatment, molecules in which no stable strand exchange has occurred will return to the high BM amplitude of the substrate; the Holliday junction and the linear excision product will retain the low BM amplitude. Similar lines of reasoning apply to recombination sites in head-to-head orientation, signifying an inversion reaction, with one important exception. In this case, the product of recombination is indistinguishable from the starting DNA substrate in its length, and hence in its BM amplitude.

Previous studies with Cre and Flp (24,25) have identified two recombinase-bound species that populate the intermediate-high BM amplitude state, a ‘non-productive complex’ that does not enter the synaptic state directly and a ‘pre-synaptic complex’ that goes on to perform synapsis (Figure 2A). A non-productive complex must dissociate and return to the substrate state before it can re-enter the recombination path to make a fresh attempt at synapsis. The low BM amplitude state is also comprised of two types of synaptic complexes (24,25). One is the ‘functional synaptic complex’ (henceforth referred to as ‘recombinogenic complex’), in which the chemical steps occur to form the Holliday junction or the recombinant product. The other is a ‘wayward synaptic complex’ (henceforth referred to as ‘wayward complex’), which does not perform stable strand exchange. Rather it reverts to the pre-synaptic state (which may then form a synaptic or a wayward complex) or to the free DNA substrate (which may start over from the step of protein association).

Regardless of site orientation (head-to-head or head-to-tail), a non-productive complex is represented by a molecule with an intermediate-high BM amplitude that switches to high BM amplitude spontaneously or upon SDS treatment (Figure 2A and B). For head-to-tail sites, a wayward complex is signified by a molecule in the low BM amplitude state that spontaneously switches to the intermediate-high (pre-synaptic state) or high BM amplitude (recombinase-free substrate state) (Figure 2A), or regains the high BM amplitude upon SDS treatment (Figure 2B). For the head-to-head sites, this phenotype may indicate either a wayward complex or a recombinogenic complex that completed recombination (DNA inversion). Retention of the low BM amplitude after SDS challenge denotes a recombinogenic complex that formed a Holliday junction in the case of head-to-head sites. This behavior in the case of the head-to tail sites indicates a recombinogenic complex that either formed a Holliday junction or completed recombination (excision) (Figure 2B).

### Thermodynamic and kinetic analyses of Cre and Flpe mutants

In the experimental analyses to follow, the TPM assays were carried out using representative point mutants of the two recombinases and DNA substrates containing a pair of the respective recombination sites in either the head-to-tail or the head-to-head orientation. Time traces of individual DNA molecules (Figure 2C; Supplementary Figure S1) after the addition of a recombinase mutant, followed by SDS challenge at the end of the incubation period, yielded the relative amounts of the aforementioned complexes formed by a mutant. The dwell times of molecules in the distinct BM amplitude states were used to derive the rate constants for the formation of individual complexes, the maturation of a complex into a downstream complex along the reaction path, or its decay into an upstream precursor complex or the recombinase-free substrate.

The relative abundance of the non-productive, wayward and recombinogenic complexes determined from previous TPM analyses of the Cre-*loxP* and Flpe-*FRT* systems (24,25) is tabulated in Figure 2D (also Table 1). The relevant kinetic constants (Figure 2E) are also listed in the top rows of Tables 2 and 3. These values provide a frame of reference for characterizing the effect of a mutation within a recombination system, and for comparing the behaviors of a pair of equivalent mutants between the two systems.

**Table 2.** Kinetic constants for Cre and pentad mutants of Cre

Recombinase/mutant	*k*_PSf_ (M^−1^ s^−1^)	*k*_RSf_ (s^−1^)	*k*_WWf_ (s^−1^)	*k*_WWd_ (s^−1^)	*k*_REC_ (s^−1^)
Cre	(6.1 ± 0.6) x 10^4^	(4.2 ± 0.7) x 10^−1^	(7.7 ± 0.6) x 10^−1^	(2.7 ± 0.5) x 10^−2^	(2.0 ± 0.1) x 10^−3^
Cre(R173A)	(1.5 ± 0.2) x 10^4^		(4.2 ± 0.2) x 10^−1^	(2.3 ± 0.2) x 10^−2^	
Cre(K201A)	(1.5 ± 0.1) x 10^4^		(10.3 ± 0.2) x 10^−1^	(4.1 ± 1.9) x 10^−2^	
			k*_WWf_ (9.3 ± 0.1) x 10^−1^	k*_WWd_ (2.6 ± 0.2) x 10^−3^	
Cre(H289A)	(1.6 ± 0.1) x 10^4^	(5.1 ± 0.3) x 10^−1^	(6.7 ± 0.4) x 10^−1^	(2.0 ± 0.3) x 10^−2^	(2.3 ± 0.3) x 10^−3^
Cre(R292A)	(1.9 ± 0.3) x 10^4^		(4.1 ± 0.2) x 10^−1^	(2.2 ± 0.3) x 10^−2^	
Cre(W315F)	(1.5 ± 0.6) x 10^4^	(6.1 ± 0.7) x 10^−1^	(8.7 ± 0.8) x 10^−1^	(1.5 ± 0.2) x 10^−2^	(1.8 ± 0.2) x 10^−3^

**Figure 3.**
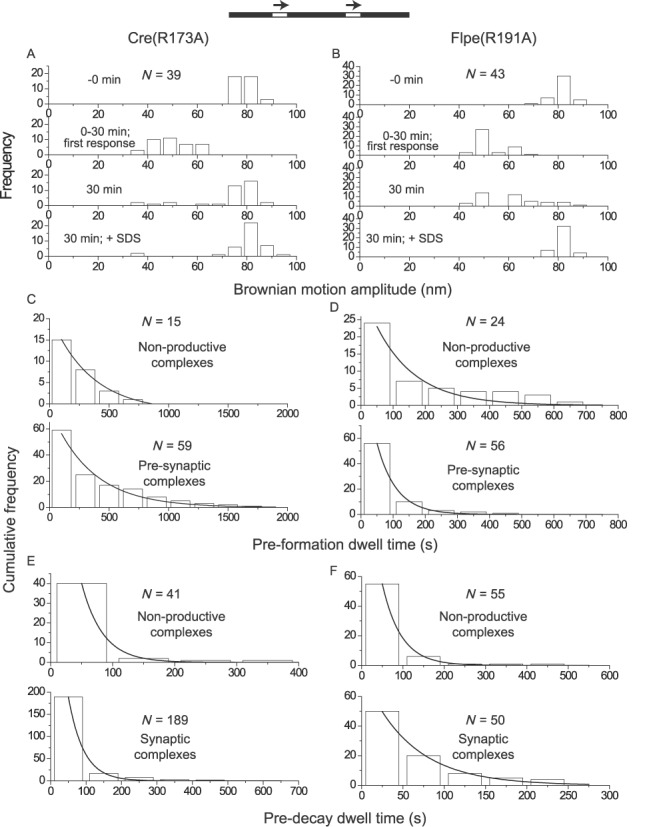
Thermodynamic and kinetic features of Cre(R173A) and Flpe(R191A). In this figure and subsequent ones (Figures 4–6), the head-to-tail orientation of the recombination sites is schematically indicated by the direction of the arrows placed above them. (**A**, **B**) The BM amplitude distributions just prior to recombinase addition (indicated as −0 min), in response to recombinase addition (0 to 30 min), at 30 min incubation, and after SDS challenge at the end of the 30 min duration are arranged from top to bottom in that order. The ‘in response to recombinase addition’ category encompasses the decreases in BM amplitude of individual DNA molecules as an immediate consequence of recombinase association, which could occur at any time during the assay (from recombinase addition to SDS challenge; 0–30 min). As a subset of the pre-synaptic associations were transient, rapidly entering synapsis, the two events could not be resolved. *N* is the number of molecules analyzed. (**C**–**F)** The dwell time histograms were fitted to a single exponential model for both Cre and Flp to derive the rate constants for the formation of non-productive and pre-synaptic complexes (*N* values refer to the number of molecules that responded to the addition of the Cre or Flp mutant) and those for the decay of non-productive and synaptic (wayward) complexes (*N* values indicate the number of events recorded by the time traces). The estimated kinetic constants are summarized in Tables 2 and 3. The rate constants for the formation and decay of the non-productive complexes by Cre(R173A) are *k*_NPf_ = (1.9 ± 0.2) x 10^4^ M^−1^ s^−1^ and *k*_NPd_ = (2.9 ± 0.4) x 10^−2^ s^−1^, respectively (not included in Table 2). The corresponding values for Flpe(R191A) are *k*_NPf_ = (4.1 ± 0.8) x 10^4^ M^−1^ s^−1^ and *k*_NPd_ = (2.2 ± 0.1) x 10^−2^ s^−1^ (not included in Table 3). The general format of this figure is maintained in Figures 4–6, representing other pentad mutants of Cre and Flpe.

**Figure 4.**
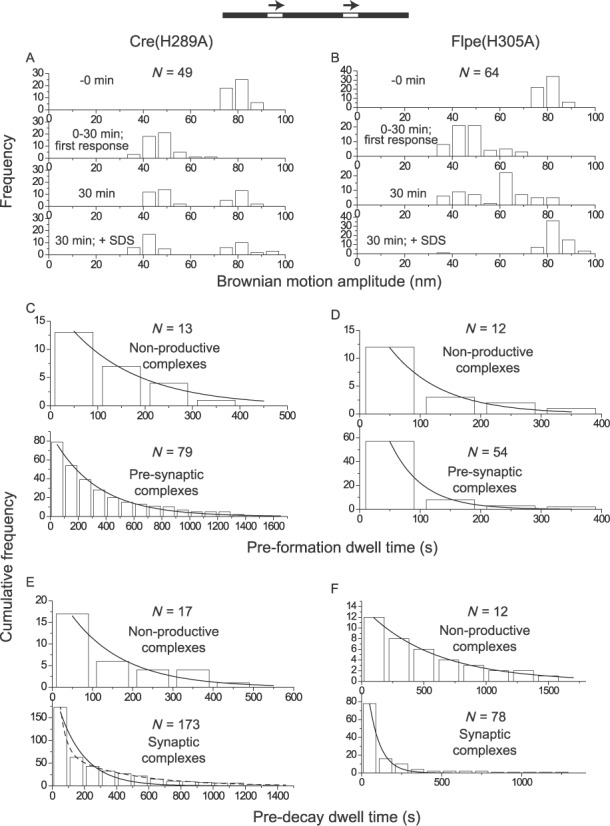
Comparison between Cre(H289A) and Flpe(H305A) by TPM characterization. **(A**, **B)** The BM amplitude histograms represent the observation of molecules over a 30 min time course, followed immediately by SDS treatment. **(C**–**F)** The dwell time histograms in C, D and F, as well as those in the top panel of E (non-productive complexes formed by (Cre(H289A)), were fitted to a single exponential model to derive rate constants for formation or decay. The dwell time histograms in the bottom panel of E (synaptic complexes formed by Cre(H289A)) were fitted to either a single exponential model (solid curve) (*R*^2^ = 0.94; *k*_1_ = (6.3 ± 0.8) x 10^−3^ s^−1^) or to a double exponential model (dashed curve). The latter accounts for the decay of the wayward complexes (*R*^2^ = 0.99; *k*_1_′ = (2.0 ± 0.3) x 10^−2^ s^−1^) as well as that of recombinogenic complexes (*R*^2^ = 0.99; *k*_2_′ = (2.3 ± 0.3) x 10^−3^ s^−1^) behaving as pseudo-wayward complexes (due to reversal of Holliday junction formation). The estimated kinetic constants are summarized in Tables 2 and 3. Not included in the tables are: *k*_NPf_ = (2.8 ± 0.1) x 10^4^ M^−1^ s^−1^ and *k*_NPd_ = (7.1 ± 0.9) x 10^−3^ s^−1^ for Cre(H289A); *k*_NPf_ = (5.4 ± 0.8) x 10^4^ M^−1^ s^−1^ and *k*_NPd_ = (1.8 ± 0.1) x 10^−2^ s^−1^ for Flpe(H305A).

**Figure 5.**
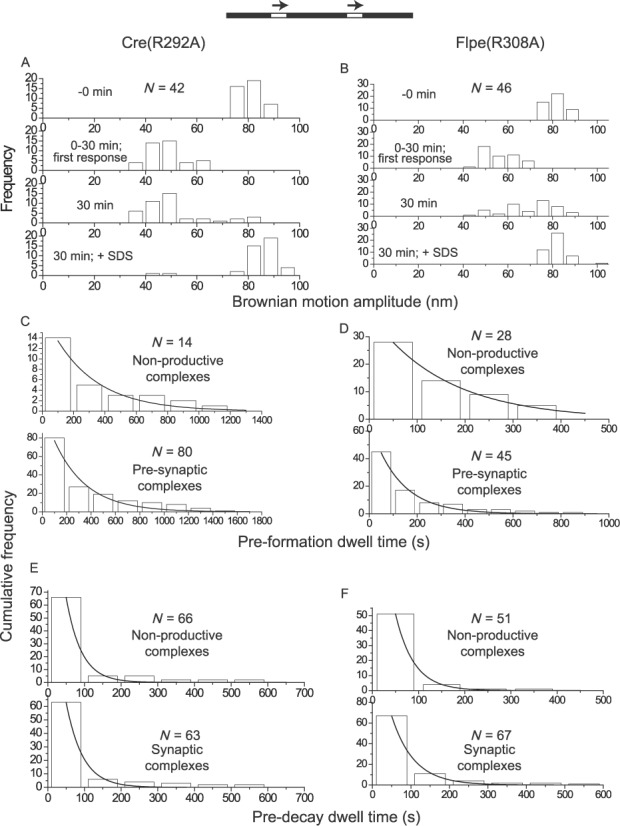
TPM behaviors of Cre(R292A) and Flpe(R308A). (**A**, **B)** The BM amplitude values of DNA molecules over a 30 min time course assay are shown by the histogram plots. **(C**–**F)** The relevant kinetic constants were derived from the dwell time histograms by single exponential fit for Cre(R292A) and Flpe(308A) (Tables 2 and 3). Not included in the tables are: *k*_NPf_ = (1.8 ± 0.3) x 10^4^ M^−1^ s^−1^ and *k*_NPd_ = (2.4 ± 0.9) x 10^−2^ s^−1^ for Cre(R292A); *k*_NPf_ = (3.2 ± 0.3) x 10^4^ M^−1^ s^−1^ and *k*_NPd_ = (2.5 ± 0.2) x 10^−2^ M^−1^ s^−1^ for Flpe(R308A).

**Figure 6.**
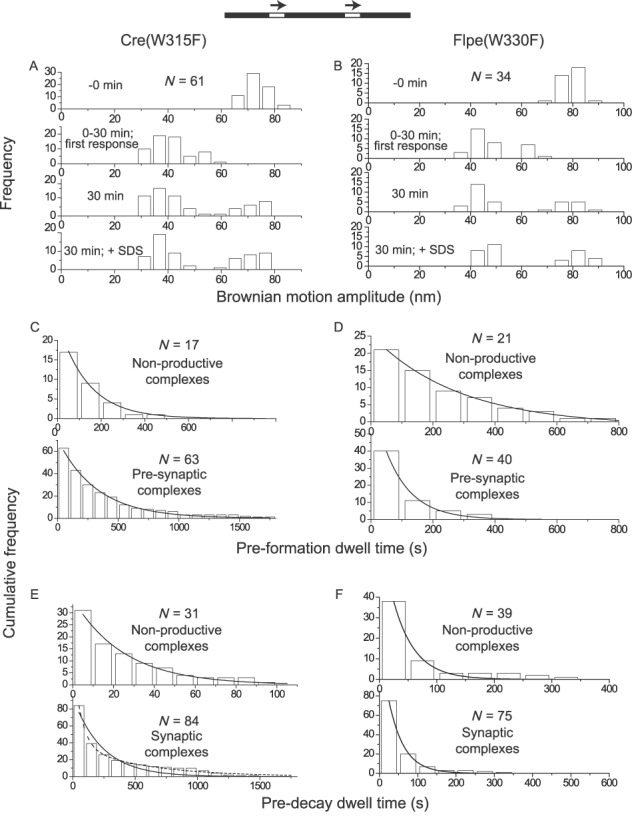
Analysis of Cre(W315F) and Flpe(W330F) by TPM. **(A**, **B)** The histogram plots were obtained from a 30 min time course assay. **(C**–**F)** For deriving kinetic constants, the dwell time histograms in C, D and F and those in the top panel of E (non-productive complexes formed by Cre(W315F)) were fitted to a single exponential model. The dwell time histograms in the bottom panel of E (synaptic complexes formed by Cre(W315F)) fitted to a single exponential model (solid curve) gave *k*_1_ = (4.3 ± 0.5) x 10^−3^ s^−1^ (*R*^2^ = 0.93). The same data fitted to a double exponential model (dashed curve) to account for reversal of the Holliday junction intermediate (as noted under Figure 4) gave *k*_1_′ = (1.6 ± 0.33) x 10^−2^ s^−1^ and *k*_2_′ = (1.8 ± 0.2) x 10^−3^ s^−1^ (*R*^2^ = 0.99) for the decay of the wayward complexes and recombinogenic complexes (by reversal of Holliday junction formation), respectively. The derived kinetic constants are summarized in Tables 2 and 3. Not included in the tables are *k*_NPf_ = (3.6 ± 0.1) x 10^4^ M^−1^ s^−1^ and *k*_NPd_ = (4.0 ± 0.3) x 10^−2^ s^−1^ for Cre(W315F); *k*_NPf_ = (2.0 ± 0.1) x 10^4^ M^−1^ s^−1^ and *k*_NPd_ = (2.7 ± 0.3) x 10^−2^ M^−1^ s^−1^ for Flpe(W330F).

The kinetic constants for Cre and Cre(K201A) were determined in previous work (24). The values for all other proteins were determined from time traces of individual molecules represented in the data shown in Figures 3–6. The rate constants for recombination (column 6) by Cre(H289A) and Cre(W315F) were obtained from the data in panel **E** of Figures 4 and 6, respectively, by fitting them to a double exponential model (Materials and Methods). An independent estimate of the rate constant for recombination by Cre(W315F) using a DNA substrate containing head-to-head *loxP* sites gave a value close to that shown here (Supplementary Figure S4). *k*_PSf_ = rate constant for the formation of pre-synaptic complexes; *k*_RSf_ = rate constant for the formation of recombination-competent synaptic complexes; *k*_WWf_ = rate constant for the formation of wayward synaptic complexes; k*_WWf_ = rate constant for the formation of long-lived wayward complexes assembled by Cre(K201A); *k*_WWd_ = rate constant for the decomposition of wayward complexes; k*_WWd_ = rate constant for the decomposition of long-lived wayward complexes formed by Cre(K201A); *k*_REC_ = rate constant for recombination (conversion of synaptic complexes into recombinant products).

**Table 3.** Kinetic constants for Flpe and pentad mutants of Flpe

Recombinase/mutant	*k*_PSf_ (M^−1^ s^−1^)	*k*_RSf_ (s^−1^)	*k*_WWf_ (s^−1^)	*k*_WWd_ (s^−1^)	*k*_REC_ (s^−1^)
Flpe	(6.0 ± 0.3) x 10^4^	(4.9 ± 0.4) x 10^−2^	(1.7 ± 0.2) x 10^−2^	(1.6 ± 0.1) x 10^−2^	(1.7 ± 0.1) x 10^−3^
Flpe(R191A)	(8.3 ± 0.4) x 10^4^		(6.1 ± 0.2) x 10^−3^	(1.8 ± 0.1) x 10^−2^	
Flpe(H305A)	(1.3 ± 0.1) x 10^5^		(8.4 ± 1.0) x 10^−3^	(1.4 ± 0.1) x 10^−2^	
Flpe(R308A)	(4.1 ± 0.3) x 10^4^		(1.0 ± 0.1) x 10^−2^	(1.7 ± 0.1) x 10^−2^	
Flpe(W330F)	(5.5 ± 0.5) x 10^4^	(2.5 ± 0.4) x 10^−2^	(2.3 ± 0.1) x 10^−2^	(2.5 ± 0.1) x 10^−2^	(2.6 ± 0.3) x 10^−3^

The analyses were performed as described in the legend of Table 2. All rate constants, except for the recombination rate constant, were determined from individual time traces corresponding to the data shown in Figures 3–6. The values for Flpe are taken from a previous study (25). The symbols for the estimated rate constants are the same as those in Table 2. The rate constant for recombination (*k*_REC_) for Flpe(W330F) was derived from assays using a substrate containing head-to-head *FRT* sites (Supplementary Figure S5).

### Arg-173 of Cre; Arg-191 of Flp

Arg-173 of Cre and Arg-191 of Flp represent the first highly conserved residue (from the amino-terminus) of the tyrosine family catalytic pentad. In the crystal structure of the Cre recombination complex, Arg-173 forms a hydrogen bond with one of the non-bridging oxygen atoms (OP1) of the scissile phosphate in the uncleaved DNA strand, and is positioned to contact both the non-bridging oxygen atoms (O1 and O2) in the cleaved strand (Figure 1A and B) (22). Furthermore, as suggested by the vanadate transition state structure, Arg-173 may assist strand cleavage by hydrogen bonding to the leaving 5′-oxygen (19). According to the Flp structure, Arg-191 contacts OP1 in the uncleaved strand and is within hydrogen bonding distance of O1 and O2 in the cleaved strand (Figure 1C and D) (32). To test whether Arg-173 and Arg-191 may influence recombination in ways other than by their direct catalytic roles suggested by structural and biochemical data (19–21), Cre(R173A) and Flpe(R191A) were subjected to TPM analysis.

Upon addition of Cre(R173A) or Flpe(R191A) to their respective DNA substrates containing head-to-tail sites, the large majority of molecules (∼83% for Cre(R173A) and ∼98% for Flpe(R191A)) (Supplementary Table S1) in the starting population responded by a downward shift in their BM amplitudes, consistent with recombinase occupancy of the target sites. The BM amplitude distributions of molecules observed over a 30 min time course, and quenched by SDS treatment at 30 min, are shown in Figure 3A and B. The dwell times in the relevant BM amplitude states provided the rate constants for the formation and decay of the corresponding complexes (Figure 3C–F; also data not shown). The relative amounts of these complexes and their kinetic features are given in Tables 1 and 2 for Cre(R173A), and Tables 1 and 3 for Flpe(R191A).

The non-productive complexes, those that failed to synapse, were ∼13% for Cre(R173A) versus ∼20% for Cre (Table 1). The corresponding values were ∼3% for Flpe and ∼26% for Flpe(R191A) (Table 1). Cre(R173A) showed a roughly 4-fold lower rate constant than Cre for the formation of the pre-synaptic complexes (*k*_PSf_), and comparable rate constants (within a factor of 2) to those of Cre for the formation (*k*_WWf_) and dissociation (*k*_WWd_) of wayward complexes (Table 2). Flpe(R191A) was slowed down in synapsis, with a rate constant ∼3-fold and ∼8-fold lower than Flpe rate constants for the formation of wayward (*k*_WWf_) and recombinogenic (*k*_RSf_) complexes, respectively (Table 3). Consistent with the catalytic defects of Cre(R173A) and Flpe(R191A), the molecules synapsed by them did not proceed beyond the wayward state.

Arg-191 of Flp promotes commitment to recombination by decreasing the fraction of non-productive complexes and increasing the rate of synapse formation. Arg-173 of Cre helps recombination by increasing the rate of assembly of pre-synaptic complexes.

### Lys-201 of Cre and Lys-223 of Flp

Previous work suggests that Lys-201 does not make a significant contribution to the binding of *loxP* sites by Cre or their synapsis, and likely serves the general acid function during strand cleavage (33,34). Lys-223 from an active Flp monomer (engaging the scissile phosphate poised for cleavage) forms a hydrogen bond with the O2 atom of the cytosine base neighboring the scissile phosphate (21). This lysine also contacts the scissile phosphate. The less well oriented Lys-223 of the inactive Flp monomer is farther from the scissile phosphate, but within contact distance of the 5′-hydroxyl group. The proximity to the leaving group would be consistent with the general acid role for Lys-223 during strand cleavage, but has not been tested.

The TPM characteristics of Cre(K201A) derived from an earlier analysis (24) are summarized in Table 1 (also Supplementary Table S1). Unlike Cre mutants containing alanine substitutions at the other pentad positions, Cre(K201A) gives synapsed complexes of two distinct types (short- or long-lived) with a ∼15-fold difference in their dissociation rate constants (Table 2). The slower one is comparable to the rate of recombination in synaptic complexes (24), even though Cre(K201A) is catalytically inactive. In the TPM assay with head-to-tail *FRT* sites, only ∼3% of the DNA molecules showed a shift in BM amplitude in response to Flpe(K223A) addition (Supplementary Table S1; data not shown), and remained as non-productive complexes (Table 1). Thus, Lys-223 of Flp comes into play at the earliest stage of recombination whereas Lys-201 of Cre functions after synapsis has been established.

In TFM-FRET measurements, approximately one-third of the synaptic complexes formed by Cre conformed to crystal structures in the distance between proximal DNA arms of paired partner *loxP* sites, while the rest assumed a distinct conformation with wider separation of these arms (27). By the same analysis, the stable complexes formed by Cre(K201A) were almost exclusively of the latter type. This alternative conformation, perhaps due to the cleavage-incompetence of Cre(K201A), may fit the long-lived wayward complexes formed by Cre(K201A) in the TPM assays (24). However, in contrast to FRET, TPM is not sensitive enough to reveal conformational differences among synapsed recombination sites.

### His-289 of Cre and His-305 of Flp

The histidine residue at the third pentad position (from the amino-terminus) is less well conserved than the arginine duo, with tyrosine or glutamine occupying this position in a small subset of the tyrosine recombinases. The crystal structures of Cre and Flp would be consistent with His-289 of Cre and His-305 of Flp assisting strand cleavage by hydrogen bonding to the scissile phosphate and/or by activating the tyrosine nucleophile (21,22). In the vanadate transition state structure of Cre, the Nϵ of His-289 is positioned to contact a non-bridging oxygen atom as well as the oxygen atom of the Tyr-324 nucleophile (19). A potential general base role for His-305 in activating Tyr-343 has been revealed in a biochemical assay (35). The absence of His-305 does not undermine strand cleavage by Flp severely. In fact, Flpe(H305L) accumulates the cleaved intermediate (36,37), presumably because of stronger impairment of the joining reaction. Consistent with a less critical role for the conserved histidine than the invariant arginines, several Cre variants containing substitutions of His-289 retain recombination activity *in vivo* and *in vitro* (19).

Addition of Cre(H289A) led to its association with ∼67% of the substrate molecules (*loxP* sites in head-to-tail orientation) (Supplementary Table S1), yielding predominantly wayward (∼73%) and recombinogenic (∼25%) complexes with a negligible fraction (∼2%) of non-productive complexes (Figure 4) (Table 1). These values are generally consistent with the observed *in vivo* and *in vitro* recombination activity of Cre(H289A) (19). The rate constant for pre-synaptic complex formation was ∼4-fold reduced for Cre(H289A) compared to Cre (Table 2). The other rate constants, including that for completion of recombination (*k*_REC_), were nearly the same for the two proteins (Table 2). Thus, in addition to its possible role in strand cleavage, His-289 kinetically promotes the formation of pre-synaptic complexes. The H289A mutation depresses overall recombination by skewing the synapsed molecules toward wayward complexes (Table 1), without affecting the kinetics of recombination.

Nearly all (∼97%) of the substrate molecules (containing head-to-tail *FRT* sites) responded to Flpe(H305A) addition (Supplementary Table S1) to form ∼14% non-productive complexes and ∼86% wayward complexes (Figure 4) (Table 1). We have verified that Flpe(H305A) is similar to previously characterized mutants at position 305 (37) in being cleavage-competent (Supplementary Figure S2). The relatively low yields of the non-productive complex from Flpe(H305A) (∼14%) compared to Flpe(R191A) (∼26%) or Flpe(R308A) (∼43%; see below) (Table 1) and the abundance of the wayward complex (∼86%) are consistent with the inference from previous studies that strand cleavage by Flp promotes synapsis of *FRT* sites (25). There was a ∼6-fold difference in the rate constants for the formation of synaptic complexes by Flpe (recombinogenic) and Flpe(H305A) (wayward) (Table 3). Thus, His-305 improves functional Flp-*FRT* association by disfavoring non-productive complexes and provides a kinetic boost to synapsis.

### Arg-292 of Cre and Arg-308 of Flp

The second arginine of the catalytic pentad is nearly invariant in the tyrosine family, with only a small number of recombinases containing a conserved substitution of lysine at this position. In the structure of the Cre-DNA complex, Arg-292 is within hydrogen bonding distance of OP2 of the scissile phosphate in the uncleaved DNA strand and with O2 in the cleaved strand (22) (Figure 1A and B). In the vanadate transition state structure, this arginine forms two hydrogen bonds, with one of the non-bridging oxygen atoms and with Tyr-324 (19). The latter interaction suggests that Arg-292 may help orient the tyrosine nucleophile and/or stabilize the developing negative charge on the phenolate moiety. In the Flp structure, Arg-308 is positioned to contact OP2 in the uncleaved strand (Figure 1C), and O2 in the cleaved strand (Figure 1D) (32). Although Arg-308 is not required for Flp binding to *FRT*, Flp-DNA interaction is weakened in its absence (37).

Approximately 88% of the DNA molecules (containing head-to-tail *loxP* sites) responded to Cre(R292A) addition (Supplementary Table S1). Non-productive complexes were ∼10%, the rest being pre-synaptic complexes that progressed to wayward complexes (Figure 5) (Table 1). There was a ∼3-fold decrease in the rate constant for pre-synaptic complex formation by Cre(R292A) compared to Cre (Table 2), suggesting that Arg-292 provides a kinetic advantage in productive Cre-*loxP* association.

Only ∼67% of the DNA molecules (containing head-to-tail *FRT* sites) responded to Flpe(R308A) (Supplementary Table S1), significantly lower than that observed for Flpe or Flpe(R191A). This decrease is consistent with the previously reported role for Arg-308 in stabilizing Flp-DNA interaction (37). There was a disproportionately high fraction of non-productive complexes (∼43%) relative to pre-synaptic complexes (∼57%), which then proceeded to form wayward complexes (Figure 5) (Table 1). The rate constant for wayward complex formation by Flpe(R308A) was ∼5-fold depressed, compared to the rate of recombinogenic complex formation by Flpe (Table 3). Thus, Arg-308 appears to help the early steps of recombination by channeling Flp-*FRT* association toward pre-synaptic complexes and by kinetically favoring synapse formation.

### Trp-315 of Cre and Trp-330 of Flp

Cre and Flp belong to the small minority of tyrosine recombinases that contains tryptophan as the final residue (carboxyl-terminal) of the catalytic pentad, with histidine occupying this position in >90% of the cases. Biochemical and structural evidence suggests that it is hydrophobicity at this position, rather than hydrogen bonding potential, that is functionally relevant in properly docking the helix bearing the tyrosine nucleophile (19,38,39). Trp-330 may play a secondary role in stabilizing the 5′-hydroxyl leaving group during strand cleavage by Flp (39).

Cre(W315A) elicited a BM amplitude response in only ∼17% of the DNA molecules containing head-to-tail *loxP* sites (Supplementary Table S1), all of which then remained as non-productive complexes (Supplementary Figure S3) (Table 1). Substrate molecules containing head-to-tail *FRT* sites did not respond to the addition of Flpe(W330A) (Supplementary Table S1; Table 1). These results are generally consistent with the behavior of these proteins in biochemical assays for recombination (19,38,39).

Roughly 61% of the substrate population responded to Cre(W315F) (Supplementary Table S1) to give ∼9% non-productive complexes and ∼65% wayward complexes, the rest (∼26%) being synaptic complexes that proceeded through strand exchange to give the Holliday junction intermediate or the excision product (Figure 6) (Table 1). The W315F mutation resulted in an increase in the assembly of wayward synaptic complexes (from ∼34% for Cre to ∼65% for Cre(W315F)) relative to recombinogenic complexes (Table 1). There was also a ∼4-fold reduction in the rate constant for the formation of pre-synaptic complexes (Figure 6C) (Table 2) due to this mutation, without a significant change in the rate constant for recombination (Supplementary Figure S4) (Table 2).

Among the ∼92% DNA molecules that responded to Flpe(W330F) (Supplementary Table S1), ∼24% formed non-productive complexes (Figure 6) (Table 1). The pre-synaptic complexes gave rise to wayward and recombinogenic complexes in roughly 1:3 molar ratio (Table 1). The W330F mutation did not significantly alter the kinetics of formation or decay of the different complexes or the kinetics of recombination (Table 3) (Supplementary Figure S5). However, the mutation induced a higher fraction of non-productive complexes and caused an increase in wayward complexes relative to recombinogenic complexes. Overall, the behavior of Flpe(W330F) in the TPM analysis is consistent with the modest reduction in its recombination potential noted in biochemical assays (38,39).

### Decay of the low BM amplitude state: kinetic distinction of Flp from Flpe(Y343F), Cre or Cre(Y324F)

The dwell times of DNA molecules with Cre-bound *loxP* sites in the low BM amplitude state, before their transition to high amplitude, are best fit by a double exponential model for both the head-to-tail and head-to-head orientation of the sites (24). This result is consistent with the chemical reversibility of the cleavage/joining reactions. For the head-to-tail sites, the double exponential represents the dissociation of the wayward complexes plus the reversal of the Holliday junction intermediate back to the parental substrate. For the head-to-head sites, the double exponential includes the dissociation of the wayward complexes as well as the inversion product resulting from recombination (forward resolution of the Holliday junction). With Flpe, a similar analysis gives a double exponential fit for head-to-head sites as expected (25). However, the results from head-to-tail sites are accommodated by a single exponential, suggesting that Holliday junction reversal is a minor process in the case of Flp. The low-to-high BM amplitude transitions thus predominantly represent the dissociation of wayward complexes.

The distinction between the Flp-*FRT* and Cre-*loxP* systems, according to the above interpretation, is absolutely dependent on strand cleavage/exchange, which is essential for the formation and resolution of the Holliday junction intermediate. It follows that the decay kinetics of the synapsed head-to-head sites must fit a single exponential model if the chemical steps are blocked by a catalytic mutation. We therefore compared the TPM behaviors of Flpe(Y343F) and Cre(Y324F) acting on head-to-head target sites.

**Figure 7.**
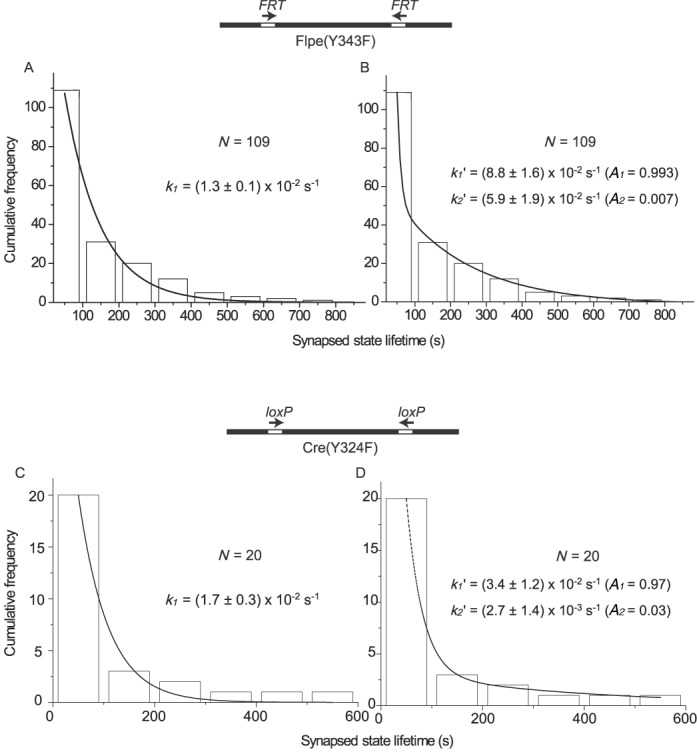
Kinetics of decay of synapsed molecules formed by Flpe(Y343F) and Cre(Y324F) in substrates containing *FRT* and *loxP* sites, respectively, in head-to-head orientation. The DNA substrates for this assay differed from those described in Figures 3–6 only in the relative orientation of the two recombination target sites (indicated schematically by the direction of the arrows). The dwell times of molecules in the synapsed state were fitted to a single exponential model (**A**, Flpe(Y343F); **C**, Cre(Y324F)) to obtain the rate constant *k*_1_ or to a double exponential model (**B**, Flpe(Y343F); **D**, Cre(Y324F)) to obtain the rate constants *k*_1_′ and *k*_2_′. *N* = the number of transition events. *A*_1_ and *A*_2_ are fitting parameters representing the relative abundance of the species corresponding to *k*_1_′ and *k*_2_′, respectively (Materials and Methods).

The low BM amplitude dwell times for Flpe(Y343F) were fit by either a single exponential (Figure 7A) or a double exponential (Figure 7B) algorithm. The single exponential model yielded a decay rate constant of (1.3 ± 0.1) x 10^−2^ s^−1^ (*R*^2^ = 0.98), close to the value ((1.7 ± 0.1) x 10^−2^ s^−1^) observed for the wayward complexes formed by wild-type Flpe (25) (Table 3). The double exponential model gave the two rate constants of (8.8 ± 1.6) x 10^−2^ s^−1^ and (5.9 ± 1.9) x 10^−3^ s^−1^ (*R*^2^ = 1.00). These were unrealistic in comparison to the estimated values for Flpe, namely, (1.7 ± 0.1) x 10^−2^ s^−1^ for wayward complex decay and (1.7 ± 0.1) x 10^−3^ s^−1^ for recombination (25) (Table 3). Furthermore, the species signified by the slower rate constant was <1% of the synapsed population (*A*_1_ = 0.993 and *A*_2_ = 0.007; Figure 7B). The transitions of synapsed head-to-head *loxP* sites formed in the presence of Cre(Y324F), according to a double exponential model, were also dominated by one process over the other (*A*_1_ = 0.97 and *A*_2_ = 0.03; Figure 7C and D). As was shown in previous studies, a single exponential model fits Flpe(Y343F) and Cre(Y324F) for synapsed head-to-tail *FRT* sites well, yielding rate constants of (1.3 ± 0.1) x 10^−2^ s^−1^ and (3.2 ± 0.3) x 10^−2^ s^−1^, respectively, for the decay of wayward complexes (24,25).

We also subjected the data in Figure 7 to unbinned MLE (maximum likelihood estimate) analysis. The single-process rate constants for Cre(Y324F) and Flpe(Y343F) were obtained as 1.3 × 10^−2^ s^−1^ (Residual = 1.0 × 10^−5^) and 8.4 × 10^−3^ s^−1^ (Residual = 1.04 × 10^−4^), respectively. The two-process rate constants for Cre(Y324F) by this method were 2.3 × 10^−2^ s^−1^ (*A*_1_ = 0.96) and 2.4 × 10^−3^ s^−1^ (*A*_2_ = 0.04) (Residual = 19.26), indicating unsatisfactory estimates. The Flpe(Y343F) data could not be fitted meaningfully (NaN = not a number) to a two-process model by MLE. Thus, by two independent methods, the decomposition of the synaptic complexes formed by Cre(Y324F) or Flpe(Y343F) is more reasonably described by a single process than by two processes.

Collectively, the kinetic data indicate that the contrasting TPM behaviors of synapsed head-to-tail sites (single exponential decay) versus head-to-head sites (double exponential decay) apply only to Flpe, and not to Flpe(Y343F) (single exponential decay in both cases), Cre or Cre(Y324F). The orientation of sites has no effect on Cre (double exponential decay) due to Holliday junction reversibility or on Cre (Y324F) (single exponential decay) due to blockage of catalysis (24).

### The geometry of the recombination synapse: effects of pentad mutations on synapse integrity

**Figure 8.**
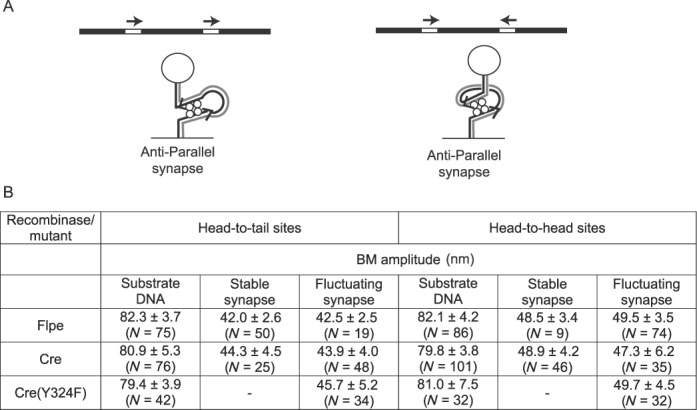
BM amplitudes of synapsed molecules containing recombination sites in head-to-tail or head-to-head orientation. **(A)** The synapsed states of head-to-tail and head-to-head sites with the sites aligned in anti-parallel geometry are schematically diagrammed at the left and right, respectively. The effective DNA tether length, which determines the BM amplitude of the attached bead, is slightly shorter for the configuration at the left. (**B)** For BM amplitude measurements, the synapsed molecules were divided into two groups, stably synapsed (BM amplitude unchanged by SDS challenge) or fluctuating (transitioning between synapsed and non-synapsed states). *N* refers to the number of protein-free substrate molecules or the number of synaptic events scored for each category.

TPM provides a sensitive probe for the geometry of the recombination synapse, as the constraint imposed on the DNA tether will be slightly different for parallel versus anti-parallel site alignment (Figure 8) (40). For a given orientation of the sites (head-to-head or head-to-tail), depending on the synapse geometry, the entry and exit points of the DNA will be either at the same end or opposite ends of the synapse (Figure 8A). The former configuration is expected to be slightly more restrictive, harbor an effectively shorter DNA tether and elicit a lower BM amplitude than the latter. For the Flp-*FRT* system, synapsed DNA molecules containing head-to-tail *FRT* sites display a lower BM amplitude than those containing head-to-head sites, consistent with the anti-parallel geometry of site alignment (40) (Figure 8B). For further verification of the predicted TPM differences resulting from the geometry of paired recombination partners, we now measured the BM amplitudes of the synapsed states of head-to-head and head-to-tail *loxP* sites in the presence of Cre and Cre(Y343F). Furthermore, to probe the potential effects of Cre and Flp pentad mutations on the integrity of the synapse, we followed the BM amplitudes of *loxP*–*loxP* synapse and *FRT*–*FRT* synapse (with sites in head-to-tail orientation) formed by these mutant proteins.

For these measurements, the synapse was classified as ‘stable’ (retaining low BM amplitude after SDS challenge) or ‘fluctuating’ (transitioning between low and high BM amplitudes). For head-to-tail sites, the stable synapse leads to exchange of one or both pairs of strands; the fluctuating synapse signifies a lack (or reversal) of strand exchange. For the head-to-head sites, the stable synapse indicates the exchange of one pair of strands (Holliday junction formation); a fluctuating synapse indicates the lack (or reversal) of strand exchange, or completion of recombination. A catalytically inactive mutant can give rise to only the fluctuating synapse. Cre(K201A), although it is blocked in the chemical steps, gives a subset of synapses that decay slower than fluctuating synapses (24) (Table 1). To distinguish them from stable synapses, they are referred to as ‘long-lived’ synapses (not resistant to SDS challenge).

The higher BM amplitude values for the synapsis of head-to-head *loxP* sites than head-to-tail sites (for the stable and fluctuating cases) are analogous to the results obtained with *FRT* sites (Figure 8B; *P* < 0.05) (40), suggesting a shared anti-parallel synapse geometry among tyrosine recombinases. Furthermore, a similar difference (depending on the orientation of the *loxP* sites) was also observed for Cre(Y324F) for the fluctuating synapse (Figure 8B; *P* < 0.05), verifying that chemical competence of the recombinase is not a pre-requisite for the selection of this geometry (34,40,41).

For the fluctuating synapse of head-to-tail *loxP* sites, a shift toward higher BM amplitudes was noticed for all the pentad mutants (*P* < 0.05), with Cre(R173A) showing the largest increase (Table 4). This was also true for the Flp mutants capable of promoting *FRT*–*FRT* synapsis (*P* < 0.05). However, in the case of the stable synapse (active in strand exchange), no significant difference was detected between the wild type and the mutants in the Cre and Flp systems. The higher BM amplitude of the stable synapse formed by the recombination-competent Cre(H289A) (*P* < 0.10) is somewhat surprising. It is possible that the Cre(H289A) synapse negotiates a non-native configuration before acquiring strand exchange capacity. Perhaps this is related to the propensity of Cre(H289A) to initiate strand exchange in a reverse order compared to Cre (42). Although Cre(K201A) is catalytically inactive, the long-lived Cre(K201A) synapse and the stable Cre synapse were not significantly different in their BM amplitudes. Except for the defect in strand cleavage, synapsis by Cre(K201A) appears to be equivalent to synapsis by Cre.

**Table 4.** BM amplitudes of the synaptic complexes formed by the pentad mutants of Cre and Flpe

Recombinase/mutant	BM amplitude (nm)		
	Head-to-tail sites		
	Substrate DNA	Stable/long-lived* synapse	Fluctuating synapse
Cre	80.9 ± 5.3 (*N* = 36)	44.3 ± 4.5 (*N* = 25)	43.9 ± 4.0 (*N* = 36)
Cre(R173A)	78.5 ± 4.2 (*N* = 76)		48.2 ± 4.1 (*N* = 31)
Cre(K201A)	78.8 ± 5.1 (*N* = 43)	45.3 ± 5.9 (*N* = 11)*	45.5 ± 5.8 (*N* = 29)
Cre(H289A)	79.6 ± 4.8 (*N* = 49)	47.0 ± 4.4 (*N* = 13)	47.1 ± 6.0 (*N* = 33)
Cre(R292A)	80.3 ± 5.4 (*N* = 42)		46.0 ± 5.9 (*N* = 35)
Cre(W315F)	80.3 ± 4.7 (*N* = 61)	42.4 ± 6.4 (*N* = 17)	46.6 ± 3.6 (*N* = 42)
Flpe	82.3 ± 3.7 (*N* = 75)	42.0 ± 2.6 (*N* = 50)	42.7 ± 2.5 (*N* = 19)
Flpe(R191A)	81.9 ± 3.7 (*N* = 73)		49.2 ± 3.1 (*N* = 33)
Flpe(H305A)	80.3 ± 4.4 (*N* = 64)		45.6 ± 5.9 (*N* = 26)
Flpe(R308A)	81.1 ± 5.5 (*N* = 46)		51.5 ± 3.1 (*N* = 26)
Flpe(W330F)	79.5 ± 3.8 (*N* = 34)	43.3 ± 3.4 (*N* = 6)	46.4 ± 2.8 (*N* = 14)

The analyses were done using a DNA substrate containing head-to-tail recombination sites. A subset of the synapsed (but wayward) complexes formed by Cre(K201A) were more stable than the fluctuating complexes (‘long-lived’; indicated by the asterisk). *N* refers to the number of substrate DNA molecules (protein-free) or the number of synaptic events from which the mean BM amplitudes were estimated.

## DISCUSSION

The present TPM analyses of the Flp-*FRT* and Cre-*loxP* systems have brought to light similarities and differences in the kinetic and thermodynamic contributions of their conserved active site pentad residues toward recombination, and in particular, toward the pre-chemical steps of the reaction (Supplementary Table S1; Tables 1–3). They are consistent with these residues playing a greater role in Flp than in Cre toward early commitment to recombination by favoring pre-synaptic complex formation over non-productive recombinase-target site association. The integrity of the recombination synapse, whose anti-parallel geometry is selected prior to strand cleavage, appears to be compromised by the lack of the pentad residues, with the possible exception of the ‘long-lived synapse’ formed by Cre(K201A).

The majority of Cre and Flp mutants tested were not seriously affected in their association with their recombination sites, although there were some differences in the amounts of the bound complex formed (Supplementary Table S1). In order to correct for these differences, the yields of non-productive, pre-synaptic and synaptic (wayward or recombinogenic) complexes in a given assay were normalized to the corresponding bound fraction.

### Roles of the catalytic pentad residues of Cre and Flp in the pre-chemical steps of recombination

The pentad residues of Flp promote early steps of recombination by promoting Flp-*FRT* association and/or lowering the fraction of non-productive complexes among Flp-bound *FRT* sites (Supplementary Table S1; Table 1). By contrast, mutations in the pentad residues of Cre, except for W315A, do not cause an increase in non-productive complexes (Table 1). The higher yield of non-productive complexes from Cre compared to the majority of pentad mutants may not be significant, as the wild type and mutant data were obtained from experiments performed at different times. Even if there is a thermodynamic cost to the conservation of certain pentad positions (Lys-201 and His-289, for example), it is a small price to pay for the enhancement in catalytic power provided by these residues. Furthermore, the dissociation of the non-productive complexes is swift relative to the time scale of recombination. They can thus be channeled back into pre-synaptic complexes by Cre more efficiently than the mutants, which are kinetically compromised in this step.

The TPM results verify and extend earlier results suggesting that the positional conservation of an active site residue need not translate into strict functional conservation as well. The pre-chemical steps proceed more or less normally in the absence of Lys-201 in Cre, but are almost completely blocked in the absence of Lys-223 in Flp. The W330A substitution has a more drastic effect on Flp-*FRT* association than W315A substitution on Cre-*loxP* association, although synapsis of the bound *loxP* sites is virtually eliminated. The H289A mutation in Cre permits recombination with reduced efficiency; the H305A mutation blocks Flp recombination after strand cleavage. The conserved arginine residues (Arg-173 and Arg-292 in Cre; Arg-191 and Arg-308 in Flp) are each non-essential for the recombinase-target site binding step, although *FRT* binding efficiency is compromised in the absence of Arg-308. Thus, the individual contributions of homologous active site residues toward their collectively conserved role in the assembly and chemical competence of the recombination complex may vary subtly between Cre and Flp.

The TPM data bring out subtle differences between Cre and Flp in the kinetic contributions of the pentad residues. In Cre, each of these residues assists recombination by increasing the rate of pre-synaptic complex formation, as revealed by phenyl alanine substitution at position 315 and alanine substitution at the other four positions (Table 2). An analogous kinetic contribution appears to be lacking in Flp, based on the behavior of Flp mutants at four pentad positions that form pre-synaptic complexes (Table 3). However, Arg-191, His-305 and Arg-308 have favorable kinetic effects on synapsis by Flp. Alanine mutations at these positions reduce (∼5 to ∼8-fold) the rate constant for synapsis (wayward) compared to that for synapsis (recombinogenic) by Flp (Table 3). The pentad residues in Cre do not seem to provide an obvious kinetic advantage in synapsis (Table 2).

### The geometry of the recombination synapse: strand cleavage in the assembly/stability of the synapse

**Figure 9.**
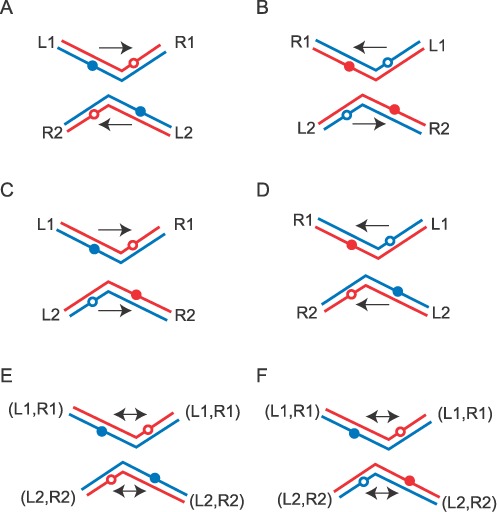
The possible geometric arrangements of partner target sites within the recombination synapse formed by Cre or Flp **(A**, **B)** The left–right orientation of the recombination sites in the DNA substrates 1 and 2 is indicated by L and R, respectively. The synapsed sites are arranged in an anti-parallel fashion (L1–R1 versus R2–L2), which is consistent with the results from this and previous studies (34,40,41). An acentric bend located close to one end or the other of the strand exchange region (with its asymmetric DNA sequence) may be responsible for selecting the synapse geometry. The DNA bend shown in A promotes cleavage and exchange of the bottom strands (blue); the bend shown in B promotes cleavage and exchange of the top strands (red). The closed circles indicate the scissile phosphodiester bonds poised for cleavage; the open circles denote those refractory to cleavage. It may not be possible to accommodate two similarly bent recombination partners (primed for either top strand cleavage or bottom strand cleavage) in a parallel fashion within the recombination synapse. (**C**, **D**) The parallel synapsis of two sites (one primed for top strand cleavage and the other primed for bottom strand cleavage) is diagrammed. However, the non-equivalence between the bends anchored at the left and right ends may preclude two such sites from forming a stable synapse. **(E**, **F**) When the sequence of the strand exchange region is symmetrized, the left–right distinction is lost, and the two types of bends become equivalent. As a result strand exchange may occur between two bottom strands (E), or between two top strands (not shown), or between a top strand and a bottom strand (F). Completion of the recombination reaction depicted in E will result in DNA deletion; that depicted in E will result in DNA inversion.

The TPM results are consistent with anti-parallel alignment of the sites in the tyrosine recombination synapse, with no requirement of strand cleavage for implementing this geometry. As suggested by the structures of Cre-DNA complexes (43), an acentric bend within the spacer region (the DNA segment sandwiched between the recombinase binding elements) may account for the selective geometry of the partner sites. Two equivalent bends, located adjacent to one spacer end or the other, will specify one of the two scissile phosphates (on the top or the bottom strand) for attack by the tyrosine nucleophile (Figure 9A and B). Accommodation of two similarly bent sites within the recombination synapse in a parallel fashion may be sterically excluded. This argument does not rule out two oppositely bent partners (one primed for top strand cleavage and the other for bottom strand cleavage) from forming a stable, if non-functional, synapse (Figure 9C and D). However, the asymmetry of the spacer sequence may induce sufficient geometric incompatibility between the two types of bends to preclude this aberrant form of synapsis. Indeed, when the spacer sequence is completely symmetrized, intra-molecular recombination between a pair of *loxP* or *FRT* sites gives inversion or deletion with roughly equal probability (44–46). In other words, there is no longer discrimination between bend orientations accommodated by the synapse, licensing the exchange of a strand from one site with either strand from the partner site (Figure 9E and F).

The present TPM analyses reveal the cleavage-inactive Flpe(R191A) and Flpe(R308A) to be competent in synapsis, with Flpe(R191A) yielding ∼77% as much synaptic complexes as Flpe (Table 1) (25). Nevertheless, among the mutants blocked in recombination, the maximum yield of wayward complexes (∼86%) is given by Flpe(H305A), which is capable of strand cleavage. Thus, cleavage may be one, but not the only, factor that promotes synapsis of *FRT* sites. In Cre, strand cleavage contributes little toward synapsis, based on the extent of wayward complexes formed by Cre(Y324F) and Cre(K201A) in a previous analysis (24) and by Cre(R173A) and Cre(R292A) in the present study (Table 1).

The higher than normal BM amplitudes of the fluctuating synapse formed by both Cre and Flp pentad mutants are consistent with modulations in the configuration and/or integrity of the synapse due to these mutations (Table 4). Flpe(R191A), Flpe(R308A) and Cre(R173A) show the maximum relative increases in BM amplitude for the synapsed states, suggesting that one or both of the conserved arginines may help the integrity of the synapse formed by tyrosine recombinases.

### Differences between Flp and Cre in reversibility

Since no exogenous supply of energy is required for recombination, the apparent lack of reversal of the intermediate steps during a Flp recombination event would seem to violate the thermodynamic symmetry of the reaction. Flp is an enzyme that catalyzes more than one round of recombination with a turnover rate of ∼0.12 per min (per Flp monomer) (47), arguing against Flp molecules being ‘spring loaded’ and acting stoichiometrically. Perhaps the conformational features of the reaction make Holliday junction formation and resolution nearly concerted events. For the head-to-tail sites, but not the head-to-head sites, the reaction would be virtually irreversible, as inferred from the TPM assays, because the circular excision product is likely to diffuse away. The estimated rate constant for recombination likely signifies the slow dissociation of a synapse in which both strand exchanges have been completed.

The difference between Flp and Cre with respect to reversibility may have a structural basis (32,48). In the Cre recombination synapse (or in the Cre-*loxP* Holliday junction intermediate), the interaction surfaces between the ‘active’ and ‘inactive’ Cre dimers are nearly the same (∼4000 Å^2^ and ∼3300 Å^2^, respectively). The corresponding interfaces for the ‘active’ and ‘inactive’ Flp dimers are significantly different (∼1600 Å^2^ and ∼930 Å^2^, respectively). The differences in the structural rigidity of the recombinase-bound Holliday junction between the two systems may account for differences in the life time of this intermediate and the likelihood for its reversal. In a biological context, a directed recombination event without reversibility is advantageous in bringing about a relevant genetic rearrangement. Additional regulatory mechanisms may confer uni-directionality to the reaction *in vivo*.

## SUPPLEMENTARY DATA

Supplementary Data are available at NAR Online.

SUPPLEMENTARY DATA
