# Supplementary material for: Single molecule TPM analysis of the catalytic pentad mutants of Cre and Flp site-specific recombinases: contributions of the pentad residues to the pre-chemical steps of recombination
Source: Nucleic Acids Res. 2015 Mar 12;43(6):3237–55. doi: 10.1093/nar/gkv114 (PMC4381057; doi:10.1093/nar/gkv114)
Supplement: SUPPLEMENTARY DATA [file supp_43_6_3237__index.html]

Single molecule TPM analysis of the catalytic pentad mutants of Cre and Flp site-specific recombinases: contributions of the pentad residues to the pre-chemical steps of recombination — Single molecule TPM analysis of the catalytic pentad mutants of Cre and Flp site-specific recombinases: contributions of the pentad residues to the pre-chemical steps of recombination — SUPPLEMENTARY DATA 

# Single molecule TPM analysis of the catalytic pentad mutants of Cre and Flp site-specific recombinases: contributions of the pentad residues to the pre-chemical steps of recombination

## SUPPLEMENTARY DATA

**Files in this Data Supplement:**

- SUPPLEMENTARY DATA
