# Supplementary material for: Single molecule TPM analysis of the catalytic pentad mutants of Cre and Flp site-specific recombinases: contributions of the pentad residues to the pre-chemical steps of recombination
Source: Nucleic Acids Res. 2015 Mar 12;43(6):3237–55. doi: 10.1093/nar/gkv114 (PMC4381057; doi:10.1093/nar/gkv114)
Supplement: SUPPLEMENTARY DATA [file supp_gkv114_nar-03417-h-2014-File015.pdf]

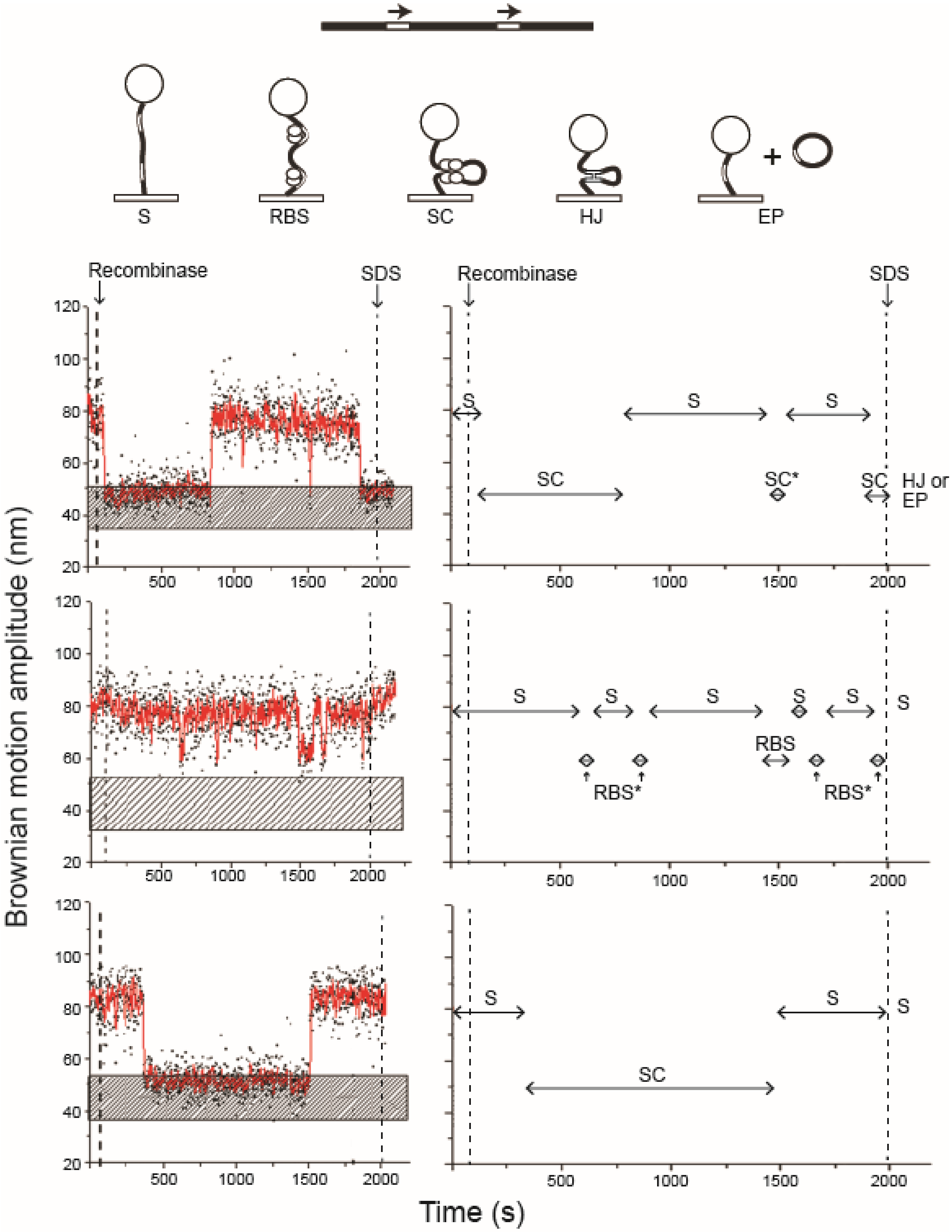

Fig. S1. Representative traces of individual molecules in different states of recombinase association. The three traces shown here were obtained with a DNA substrate containing head-to-tail recombination sites. In this figure, and also subsequent ones, the relative orientation of the

sites is schematically indicated by the direction of the arrows placed above them. The recombinase-free and recombinase-associated states of the DNA substrate, the Holliday junction intermediate and the excision products are represented as in Fig. 2. The dashed lines at the left and right of each trace indicate the additions of the recombinase and SDS, respectively. The dynamics of DNA in response to recombinase association conveyed by a trace are schematically diagrammed in the corresponding right panel. The horizontal stippled bar marks the BM amplitude of the synapsed state. **A**. Recombinase association led to early synapsis of the bound sites in this molecule. However, the synapsis dissociated subsequently to the free DNA state. This synaptic complex was therefore a wayward complex. Formation of second highly transient wayward synapse is suggested by the downward spike in the trace at ~1500 s. The molecule again formed a synaptic complex just before protein dissociation by SDS. The retention of post-SDS low BM amplitude indicates that this synaptic complex was recombinogenic, promoting formation of the Holliday junction or the excision products. **B**. This DNA molecule formed a short-lived non-productive complex at least once, which, rather than forming a synapse, dissociated to the protein-free substrate. Additional sharp dips in the trace (~625 s; ~875 s; ~1650 s; immediately before SDS addition) might indicate rapidly dissociating non-productive complexes. **C**. The molecule in this trace formed a wayward synaptic complex, and dissociated without successful completion of strand exchange. S = Substrate. RBS = Recombinase-bound substrate. SC = Synaptic complex. HJ = Holliday junction. EP = Excision products. The asterisk denotes a change in the Brownian motion (BM) amplitude consistent with the formation of a highly unstable complex.

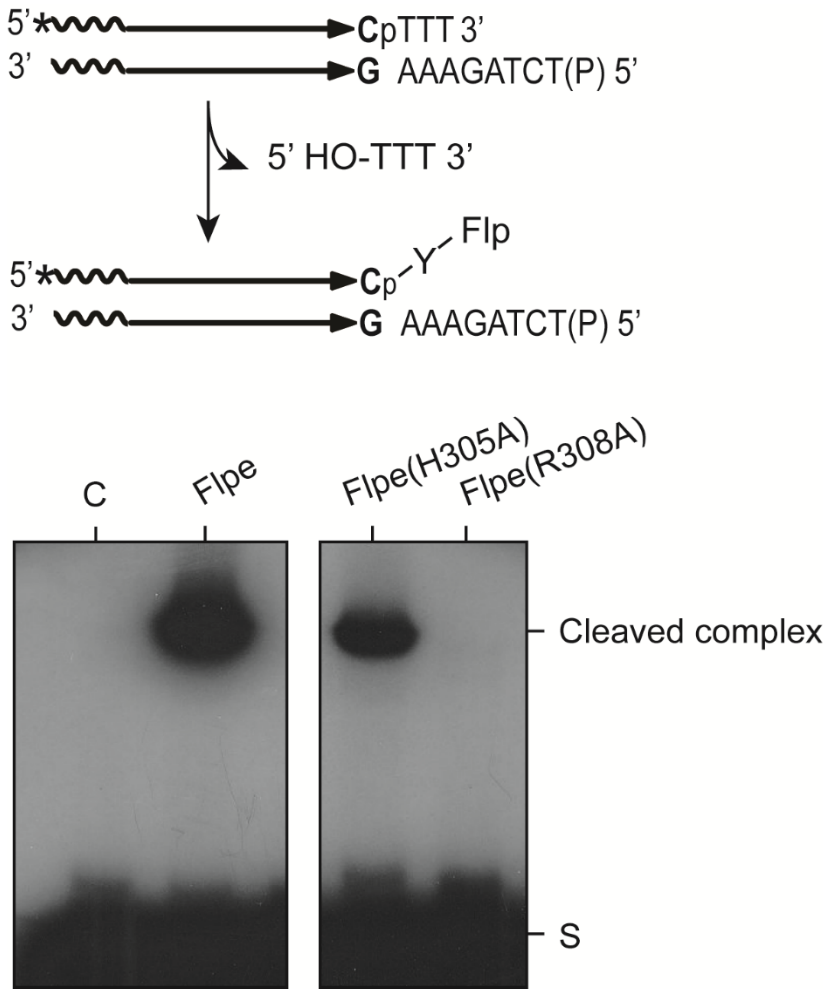

Fig. S2. Flpe(H305A) is competent in DNA cleavage. The half-site DNA substrate used for the cleavage assay is schematically diagrammed at the top. The Flp binding element is shown by the horizontal arrows, with its terminal CG bp immediately flanking the strand exchange region in bold letters. The wavy lines indicate non-specific sequences. The substrate was labeled at the 5'-end of the top strand with P^{32} (shown by the asterisk). Following cleavage and covalent attachment of Flp to the scissile phosphate (p), the trinucleotide TTT will diffuse away from the reaction center. The 5' end of the bottom strand was phosphorylated to block it from attacking the phosphotyrosyl bond present in the cleaved intermediate in a pseduo-joining reaction. The

cleavage reactions were fractionated by electrophoresis in a 10% SDS-polyacrylamide (acrylamide to bis-acrylamide, 1:29). The covalent protein-DNA adduct (cleaved complex) and the uncleaved substrate (S) were detected by phosphorimaging. A control reaction without addition of Flpe or a Flpe mutant is indicated by ‘C’. Flp(R308A), lacking cleavage activity, was included in the assay as an additional negative control.

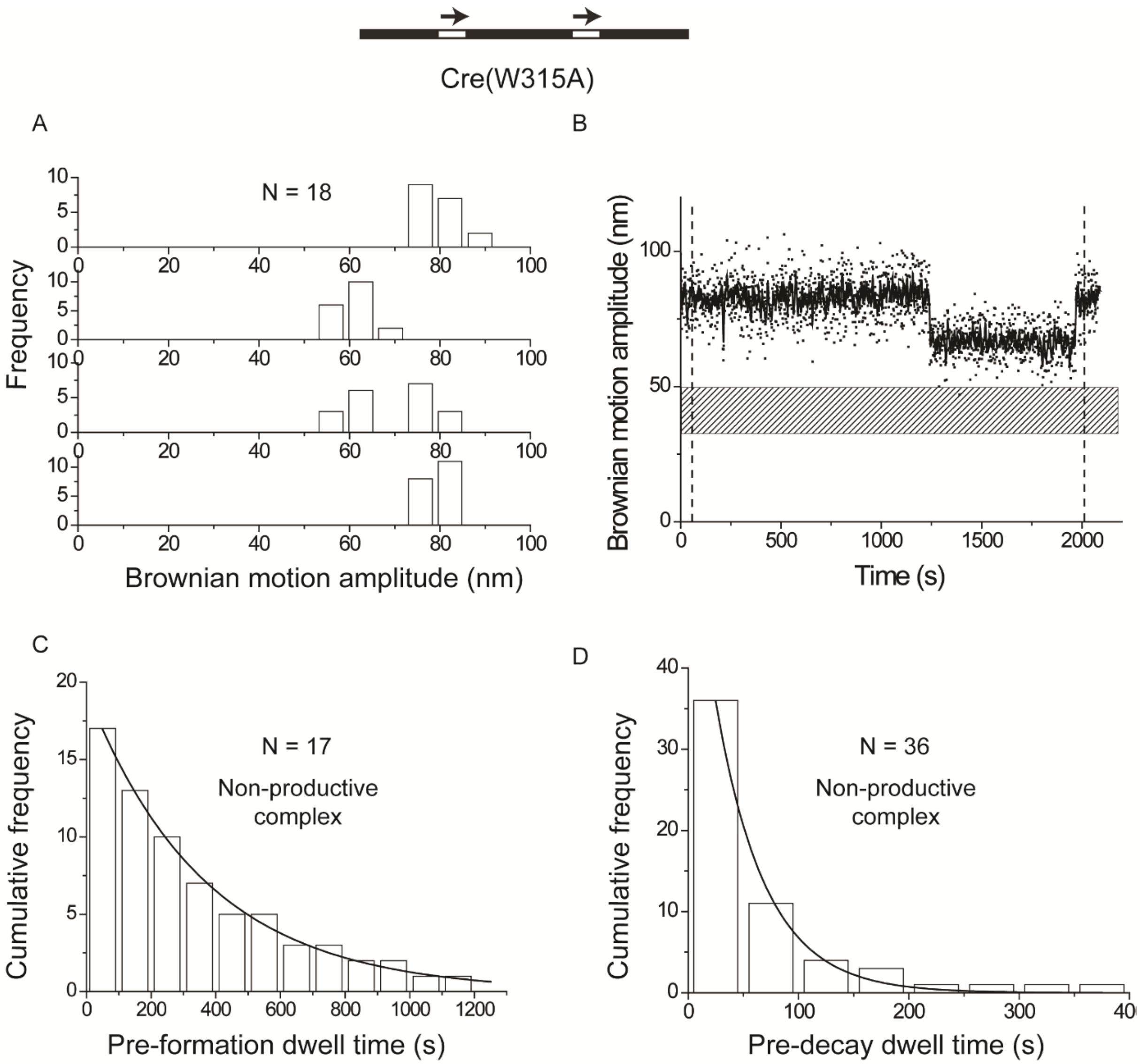

Fig. S3. The TPM analysis of the association of Cre(315A) with a DNA substrate containing *loxP* sites in head-to-tail orientation. The DNA substrate employed for the assays depicted in Fig. 3-6 was employed here as well. **A**. The BM amplitude distributions immediately before the addition of Cre(W315A) indicated as -0 min, in response to its addition (0 to 30 min), at 30 min incubation, and after SDS challenge at the end of this incubation period are arranged from top to bottom in that order. N is the number of molecules analyzed. **B**. The time trace of a single molecule that

formed a non-productive complex is shown as an example. All the Cre(W315A)-bound molecules showed a similar pattern of behavior. The dashed lines at the left and the right indicate the time of addition of Cre(W315A) and SDS, respectively. **C**, **D**. The dwell time histograms were fitted to a single exponential model to obtain the rate constants for the formation and decay of non-productive complexes as k_{NPf} = (1.4 ± 0.04) x 10^4 M^{-1} s^{-1} (R^2 = 0.98) and k_{NPd} = (2.5 ± 0.2) x 10^{-1} s^{-1} (R^2 = 0.99), respectively. N refers to the number of events recorded by the time traces.

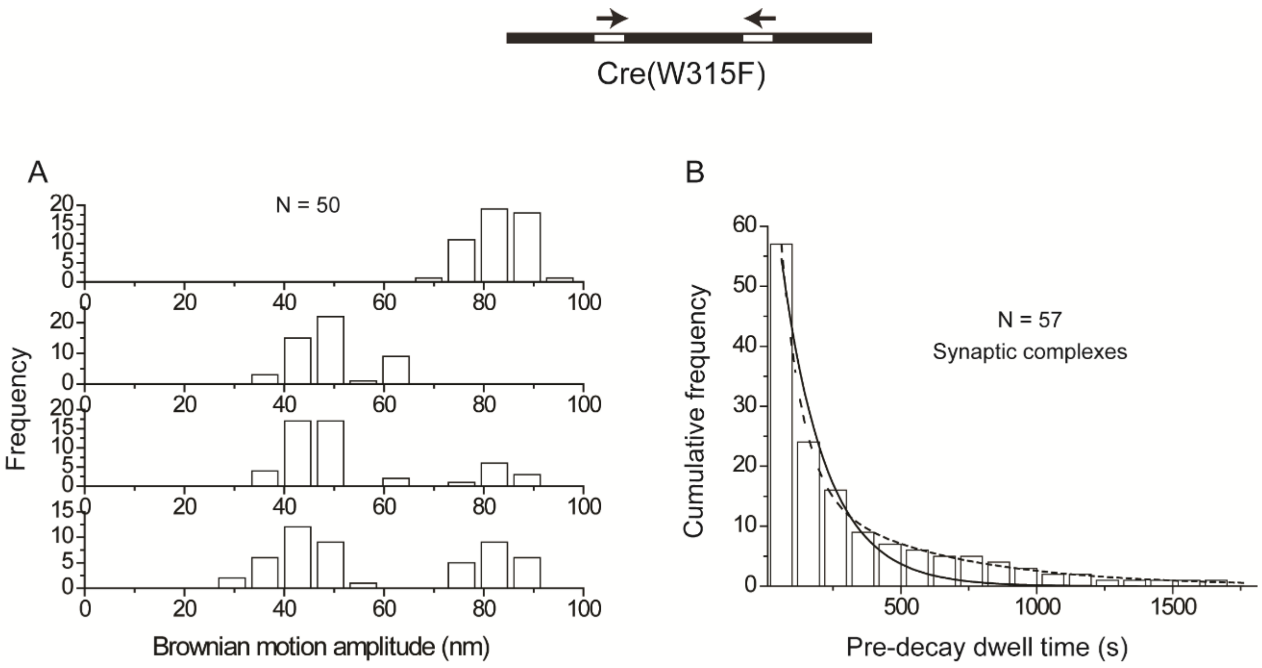

Fig. S4. The interactions of Cre(W315F) with a DNA substrate containing *loxP* sites in head-to-head orientation. Except for the change in the relative orientation of the *loxP* sites, the substrate was identical in its length and the location of the *loxP* sites to that employed in the assays shown in Fig. 3-6 and Fig. S3. **A**. The BM amplitude distributions prior to and following the addition of Cre(W315F) are arranged from top to bottom as in Fig. S3. N is the number of molecules analyzed. **B**. The pre-decay dwell times in the synapsed state were fitted to a double exponential model (dashed curve) to obtain the rate constant for the decay of the wayward complexes (k_{WWd} = (1.3 ± 0.1) x 10^{-2} s^{-1}; R^2 = 1.0) and that for recombination (k_{REC} = (2.0 ± 0.2) x 10^{-3} s^{-1}; R^2 = 1.0). Fitting the same data to a single exponential model (solid curve) gave a rate constant k = (6.1 ± 0.5) x 10^{-3} s^{-1} (R2 = 0.96). The number of low to higher amplitude transition events, N, were obtained from the time traces of individual molecules.

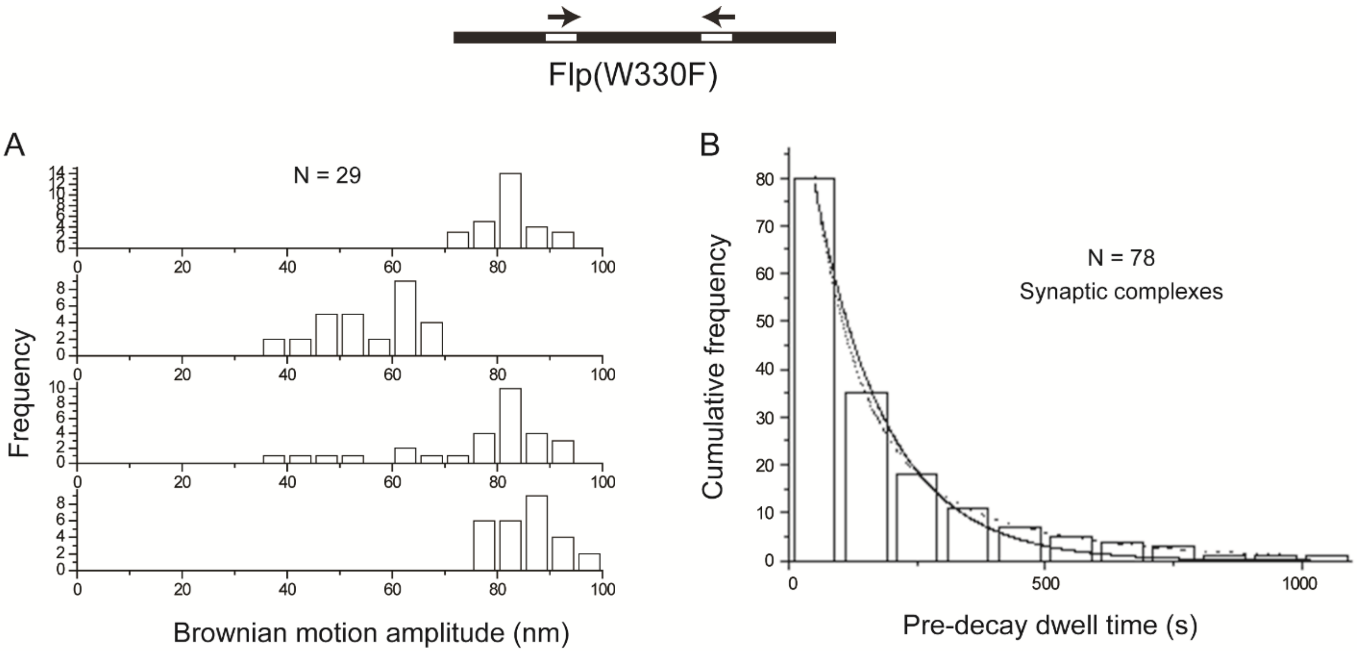

Fig. S5. Association of Flpe(W330F) with a DNA substrate containing *FRT* sites in head-to-head orientation. The DNA substrate for this assay was the same as that described under Fig. 7. The analysis was performed as in Fig. S3 and S4, and the results are presented similarly. N = number of molecules analyzed. **B**. By fitting the dwell time histograms to a double exponential model (dashed curve), the following rate constants were obtained: $k_{WWd} = (1.3 \pm 0.1) \times 10^{-2}\ s^{-1}$ ($R^2 = 1.00$) and $k_{REC} = (2.6 \pm 0.3) \times 10^{-3}\ s^{-1}$; ($R^2 = 1.00$). A single exponential mode (solid curve) gave a rate constant of $k = (7.0 \pm 0.6) \times 10^{-3}\ s^{-1}$ ($R^2 = 0.97$). N = number of transition events from individual time traces.

Recombinase/ mutant	DNA–association (%)
Cre	89.7
Cre(R173A)	83.3
Cre(K201A)	89.5
Cre(H289A)	67.3
Cre(R292A)	88.4
Cre(W315A)	16.5
Cre(W315F)	60.6
Cre(Y324F)	67.5

Recombinase/ mutant	DNA–association (%)
Flpe	98.1
Flpe(R191A)	97.5
Flpe(K223A)	< 3.0
Flpe(H305A)	97.1
Flpe(R308A)	66.9
Flpe(W330A)	ND
Flpe(W330F)	92.4
Flpe(Y343F)	48.7

Table S1. Association of Cre and Flp with their respective target sites. The association of a recombinase with a DNA molecule was registered by a decrease in the BM amplitude from that prior to recombinase addition. The initial association gave non-productive or pre-synaptic complexes, the latter often rapidly forming synaptic complexes (wayward or recombinogenic), with an additional decrease in the BM amplitude. The fraction of DNA molecules that were bound by wild type Cre or Flp and each of the indicated pentad mutants is listed. In a given assay, the bound fraction was assigned a value of 100% for quantifying its transitions into distinct types of pre-chemical complexes (Table 1). 'ND' indicates that recombinase-DNA association, as

measured by the BM amplitude decrease, was not detected. Note that recombinase occupancy of only one of the two binding elements of the target sites or their full occupancy by a recombinase mutant incompetent for DNA bending will not produce the predicted drop in BM amplitude. Such events will therefore be missed in the TPM assay.
